# Study on the Measurability of Gear Analytical Parameters in Double-Flank Measurement

**DOI:** 10.3390/s23249728

**Published:** 2023-12-09

**Authors:** Xiaoyi Wang, Mingkang Liu, Tianyang Yao, Kunlei Zheng, Chengxiang Zhao, Longyuan Xiao, Dongjie Zhu

**Affiliations:** 1 School of Mechatornics Engineering, Henan University of Science and Technology, Luoyang 471003, China; kklmk163@163.com (M.L.); yty0101@139.com (T.Y.); zkl15538694420@163.com (K.Z.); zcx_98@126.com (C.Z.); xly15137615751@163.com (L.X.); zdjwsbn@163.com (D.Z.); 2 Henan Key Laboratory of Mechanical Design and Transmission System, Henan University of Science and Technology, Luoyang 471003, China

**Keywords:** gear metrology, double-flank measurement, analytical parameters, measurable area, profile

## Abstract

Double-flank measurement is the most commonly used full inspection method on the shop floor. However, the double-flank measurement method cannot measure analytical parameters such as pitch deviations and profile deviations, and this limitation is a pain point in the field of gear measurement. This paper studies the measurability of the analytical parameters of gears based on the results of double-flank measurement, proposes the definition of measurable area, and gives the relationship between the size of the measurable area and the number of teeth and the pressure angle and the gear error. Digital simulation methods were used to conduct measurement experiments on gear analytical parameters. In the experiments, the measurability of the analytical parameters of gears with various typical profile deviations in the double-flank measurement process was verified and analyzed. The test results show that not all profile deviations are unmeasurable in the process of double-flank measurement, but there exists a profile region in which the analytical parameters of the gear can be measured accurately. The size of the measurable area of the profile is mainly determined by the number of teeth and pressure angle of the gear, while the pitch deviations are always measurable under normal conditions.

## 1. Introduction

Mass-produced automobile gears and household product gears are irreplaceable in the national economy and national defense construction. Gear measurement plays an important role in gear quality control. Gear measurement can be categorized into composite measurement and analytical measurement. Analytical measurement is mainly used to acquire pitch deviations, profile deviations, and helix deviations, which are mainly used to analyze the source of process error. Meanwhile, composite measurement is mainly used to judge whether the product is qualified [1,2].

For the quality control of mass-produced gears, the double-flank composite test is the most commonly used inspection method [3,4]. In the field of gear measurement, it is generally believed that based on gear double-flank measurement, analytical parameters, such as pitch deviations, profile deviations, and helix deviations, cannot be obtained, and only radial composite parameters can be obtained [5,6,7]. If the analytical parameters can be obtained based on gear double-flank measurements, the double-flank measurement will be much more valuable, and both low cost and high performance will be realized, which can control the quality of gears well, reduce the cost of inspection, and increase the competitiveness of the enterprise’s products.

The literature [8] improves the gear selecting machine by the use of the gear double-flank meshing measurement principle to measure parameters such as gear defects and runout. The literature [9] analyzes the effect of the radial deformation of gears under the action of measuring force in double-flank measurement and proposes relevant algorithms to enhance the accuracy of the evaluation. The literature [10] developed a cloud processing system for gear double-flank meshing measurements to improve the utilization of information. In addition, there is a large amount of literature related to the measurement of double meshed gears, mainly focusing on how to improve the measurement accuracy and measurement efficiency [11,12,13,14,15]. Aiming at obtaining analytical parameters such as pitch deviations and profile deviations from gear double-flank measurement, some scholars have carried out research from different perspectives, and some progress has been made.

References [16,17] propose a method that uses a single tooth rack probe for radial comprehensive measurement, which can obtain the tooth profile deviations and pitch deviations of the left and right flanks of the tested gear, providing a way to measure the multiple parameters of gear accuracy simultaneously. Through further research on double-flank measurement based on rack-type probes, the literature [18] describes the double-flank rack probe (DFRP) method for the measurement of pitch and profile deviations of left and right tooth flanks and develops a gear measurement device based on the DFRP method. The literature [7] proposes a double-flank multi-dimensional measurement principle for gears, and an on-line gear measuring machine developed based on this principle can simultaneously obtain the radial comprehensive deviation and helical deviations of the measured gear. The models of radial and tangential errors in the double-flank test process were established in the literature [19], and the profile deviations and pitch deviations imported by the base circle radius deviations were simulated and measured based on the rack probe.

However, in the above studies, due to the use of rack-type probes or special probes, a conventional double-flank rolling tester cannot be used, and specialized measuring instruments need to be developed. The use of a single tooth rack-type probe cannot achieve continuous rotation measurement (like the conventional double-flank rolling test), and the efficiency is low when measuring gears with a large number of teeth. In conclusion, the existing double-flank rolling tester using a gear-shaped master gear cannot obtain analytical parameters such as pitch deviations and profile deviations.

In this paper, a new structure of the double-flank rolling tester is proposed, which adds two angle sensors to the conventional double-flank rolling tester and utilizes the master gear as a probe, which can realize the measurement of analytical parameters such as pitch deviations and partial profile deviations.

Firstly, based on the new double-flank rolling tester, this paper analyzes the measurability of analytical parameters in double-flank measurement. Secondly, the relationship between the basic parameters of the gear pair, profile deviations, and the size of the profile region of the measurable analytical parameter is explored. Finally, the measurement test of the analytical parameters in double-flank measurement was carried out by using the simulation method, which verified the measurability of the analytical parameters in double-flank measurement under a variety of profile deviation conditions.

## 2. Measurability Analysis

In order to achieve the measurement of analytical parameters based on the double flank, two angle sensors need to be added to the conventional double-flank instrument with some changes in the structure of the instrument. On the basis of the instrument, the measurability of the analytical parameters of the double-flank measurement using the master gear is explored, and a calculation method for the size and position of the profile area in which the analytical parameters can be obtained in the process of double-flank meshing of the gear is attained. Further, the relationship between the size of the measurable area of the analytical parameters of the product gear and the number of teeth and pressure angle of gears, as well as the relationship between the measurable area of the analytical parameters and the profile error, was explored and obtained.

### 2.1. Instrument

**Figure 1:**
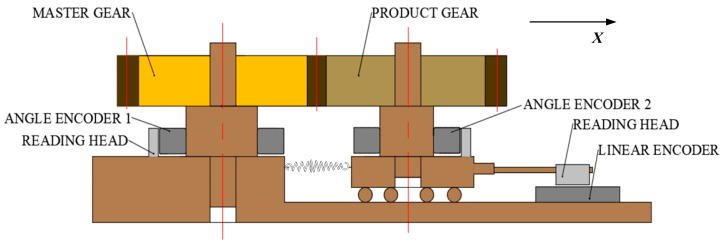
Schematic diagram of a double-flank rolling tester for obtaining analytical parameters.

Figure 1 shows a double-flank rolling tester that allows analytical parameters to be obtained. Unlike a conventional double-flank tester, the new tester is equipped with an angle measuring system composed of two angle encoders attached to two axes of a master gear and measured gear, respectively. As with the conventional double-flank rolling tester, there is a spring loading device and a sensor for measuring the center distance. Compared with a conventional double-flank rolling tester, the fit between the shaft and the gear has been changed from the clearance fit to the tight fit. In order to increase the efficiency of gear replacement, it is an option to use an expandable arbor to fix the product gears.

In the measurement device shown in Figure 1, the master gear drives the angle encoder 1. The product gear rotates coaxially with the angle encoder 2 and shifts along the *X*-axis. The rotation angles are measured by two angle encoders, and the *X*-axis displacement is measured by a linear encoder. Based on the information on the two angles and one displacement measured by three sensors, the gear analytical parameters can be obtained, and then the gear accuracy grade of the corresponding parameters can be evaluated.

### 2.2. Analysis

In this paper, the measurability of analytical parameters of involute cylindrical gears is studied. The analytical methods for other types of gears are similar.

According to the gear geometry, the transverse base pitch *Pb* and the contact ratio *ε* of double-flank meshed gear pairs can be obtained from Equations (1) and (2),

(1) $$Pb=\pi m\cdot \cos\left(\alpha_{0}\right)$$

(2) $$\varepsilon =\left(z_{1}\cdot \left(\tan\left(\alpha_{a1}\right)-\tan\left(\alpha_{0}\right)\right)+z_{2}\cdot \left(\tan\left(\alpha_{a2}\right)-\tan\left(\alpha_{0}\right)\right)\right)/\left(2\pi \right)$$

where $\alpha_{0}$ is the transverse pressure angle, *m* is the modulus, and *z*_1_ and *z*_2_ are the number of teeth on the master gear and the product gear, respectively. $\alpha_{a1}$ and $\alpha_{a2}$ are the pressure angles on the tip diameter of the master gear and the product gear, respectively.

**Figure 2:**
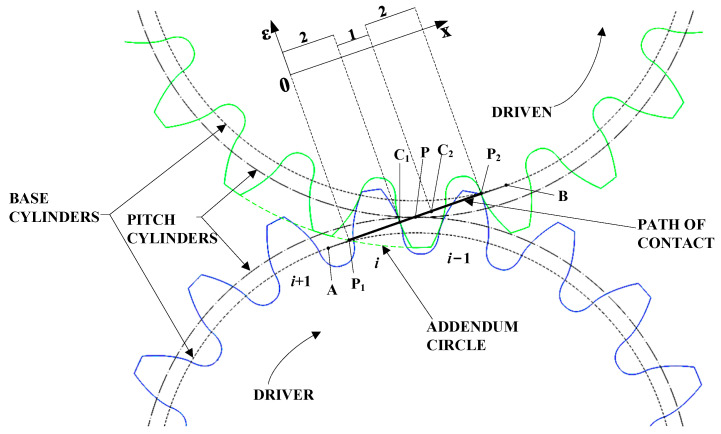
Relation between the path of contact and instantaneous contact ratio of gears.

As shown in Figure 2, taking the gear parameters in Table 1 as an example, the master gear is the driving gear and the product gear is the driven gear, and both gears are standard gears; the contact ratio is less than 2. Points A and B on the path of contact are the tangent points between the path of contact and the base circle of the master gear and the product gear, respectively. Points P_1_ and P_2_ are the intersection of the tip circle of the product gear with the path of contact and the intersection of the tip circle of the master gear with the path of contact, respectively. Points C_1_ and C_2_ are the demarcation points where the instantaneous contact ratio changes during the meshing process. Point P is the pitch point of the two gears.

From the geometric relationship in Figure 2 and the definition of contact ratio, it can be concluded that

(3) $$\{\begin{array}{c} P_{2}C_{1}=P_{1}C_{2}=Pb\\ P_{1}P_{2}=Pb\cdot \varepsilon \end{array}$$

where P_2_C_1_, P_1_C_2_, and P_1_P_2_ are the lengths of the corresponding line segments on the path of contact in Figure 2. From the geometrical relationship in Figure 2 and Equation (3), the following can be calculated:

(4) $$P_{1}C_{1}=P_{2}C_{2}=P_{1}P_{2}-P_{2}C_{1}=Pb\cdot (\varepsilon -1)$$

where P_1_C_1_ and P_2_C_2_ are the lengths of the corresponding line segments on the path of contact in Figure 2. Then

(5) $$C_{1}C_{2}=P_{2}C_{1}-P_{2}C_{2}=Pb\cdot (2-\varepsilon )$$

As shown in Figure 2, during the double-flank meshing process, the number of pairs of teeth in instantaneous meshing will change regularly. From Equations (4) and (5), the regions P_1_C_1_ and P_2_C_2_ on both sides of P_1_P_2_ are the corresponding double-flank meshing regions [20], where the instantaneous contact ratio of the corresponding flanks is two. When the actual meshing points are located in the region of P_1_C_1_ and P_2_C_2_, the gear double-flank measurements are obtained as a result of the combined profile deviations of corresponding flanks on both teeth. Then, the profile regions corresponding to the P_1_C_1_ and P_2_C_2_ are defined as “unmeasurable areas”. The region C_1_C_2_ is the single corresponding flank meshing region, where the instantaneous contact ratio of corresponding flanks is one. When the instantaneous meshing point is within C_1_C_2_, the deviations of the gear double-flank measurements are determined by the profile deviations of a single tooth flank, so the measured error directly reflects the profile deviations of the flank. The profile regions corresponding to C_1_C_2_ are defined as “measurable areas”. In this area, the profile deviations are obtainable, and then the pitch deviations can be calculated.

**Figure 3:**
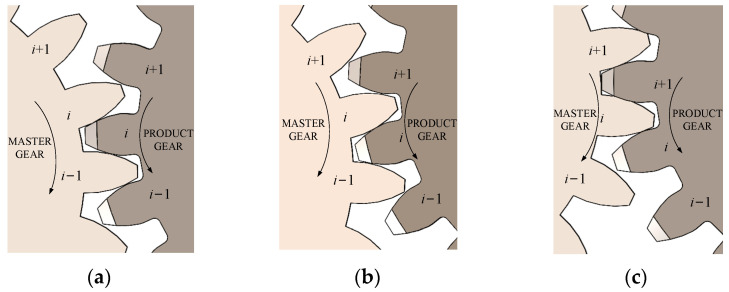
Schematic diagram of gear meshing states. (**a**) State 1: the instantaneous contact ratio of right flank is 2. (**b**) State 2: the instantaneous contact ratio of right flank is 1. (**c**) State 3: the instantaneous contact ratio of right flank is 2.

Figure 3 is a schematic diagram of the three states of the instantaneous contact ratio variation. In Figure 3, the product gear meshes with the master gear sequentially at the *i* − 1th, *i*th, and *i* + 1th teeth. The master gear is the driving gear and rotates clockwise. The product gear is the driven gear and rotates counterclockwise. Figure 3a shows the state in which the right tooth flanks of the *i*th tooth and the *i* − 1th tooth of the master gear are engaged at the same time, which corresponds to the P_1_C_1_ region in Figure 2. Figure 3b shows the state where only the right tooth flank of the *i*th tooth participates in meshing in the right tooth flank of the master gear, which corresponds to the C_1_C_2_ region in Figure 2. Figure 3c shows the state in which the right tooth flanks of the *i*th tooth and the *i* + 1th tooth of the master gear engage at the same time, which corresponds to the P_2_C_2_ region in Figure 2.

**Figure 4:**
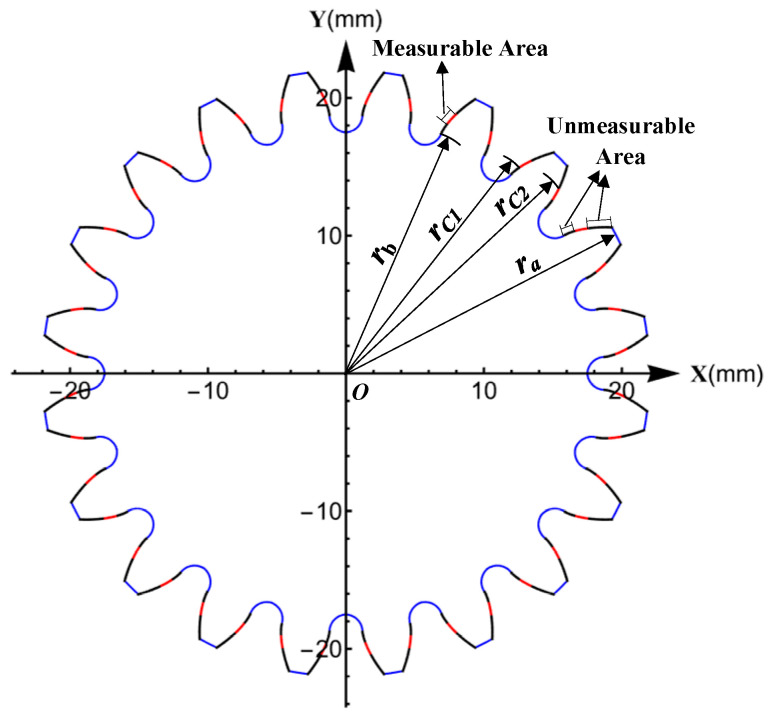
The distribution of measurable and unmeasurable areas on the profiles of the product gear.

Taking the parameters of the master gear and the product gear shown in Table 1, according to the above definitions and Equations (6) and (7), the range of the measurable area can be calculated, as shown in Figure 4.

(6) $$\{\begin{array}{c} BC_{1}=BP_{1}-P_{1}C_{1}=r_{b}\cdot \tan(\alpha_{a2})-Pb\cdot (\varepsilon -1)\\ BC_{2}=BP_{1}-P_{1}C_{1}-C_{1}C_{2}=r_{b}\cdot \tan(\alpha_{a2})-Pb\cdot (2-\varepsilon )-Pb\cdot (\varepsilon -1) \end{array}$$

(7) $$\{\begin{array}{c} \alpha_{C1}=\arctan(\frac{AC_{1}}{r_{b}})\\ \alpha_{C2}=\arctan(\frac{AC_{2}}{r_{b}}) \end{array}$$

where BC_1_, BC_2_, and BP_1_ are the lengths of the corresponding line segments on the path of contact in Figure 2, *r*_b_ is the radius of the base circle of the product gear, and $\alpha_{C1}$ and $\alpha_{C2}$ are the pressure angles of the product gear corresponding to points C_1_ and C_2_, respectively.

From the above equations, the pressure angle range of the measurable area is (16.398°, 23.531°), as shown in Figure 4. The unmeasurable area is divided into two parts, which are on both sides of the measurable area.

### 2.3. Geometric Factors Affecting the Measurable Area

The size of the measurable area of the analytical parameters is related to the basic parameters of the master gear and the product gear. The number of teeth and the pressure angle are the main parameters that affect the size of the measurable area. In transmission, the modulus of two gears is usually equal, and the change in the modulus is equivalent to proportionally increasing or decreasing the size of the gear. Therefore, the module is not the main parameter that affects the size of the measurable area.

The proportion of the measurable area to the entire meshing area of profile can be calculated with reference to the radius or with reference to the roll length. In this paper, the proportion of the measurable area (P_ma_) over the entire profile meshing region is defined as

(8) $$P_{\mathrm{ma}}=\left(r_{C2}(z_{1},z_{2},\alpha_{0})-r_{C1}(z_{1},z_{2},\alpha_{0})\right)/\left(r_{a}-r_{b}(z_{2},\alpha_{0})\right)$$

where $r_{C1}$ and $r_{C2}$ are the radius of the points on the profile of the product gear corresponding to points C_1_ and C_2_ in Figure 2, $r_{a}$ is the radius of the tip circle of the product gear, and *r_b_* is the radius of the base circle of the product gear. Among them, $r_{C2}$ and $r_{C1}$ change with $z_{1},z_{2}$ and $\alpha_{0}$, $r_{b}$ changes with $z_{2}$ and $\alpha_{0}$.

**Figure 5:**
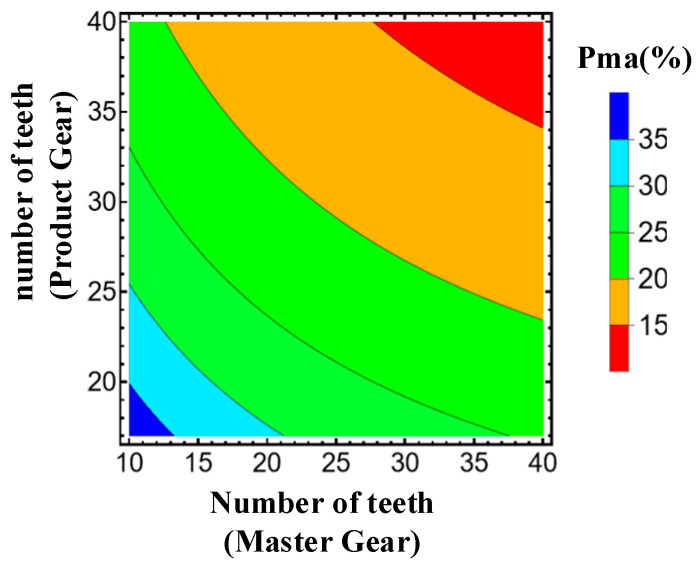
Relationship between the number of teeth and the proportion of measurable area ($\alpha_{0}$ = 20°).

The relationship between the number of teeth and the proportion of measurable area is shown in Figure 5. The P_ma_, between 15% and 35%, corresponds to the variation in the number of teeth of the master gear between 10 and 40 and the variation in the number of teeth of the product gear between 17 and 40. The percentages are larger when the number of teeth is smaller. The maximum percentage occurs at $z_{1}$ = 10 and $z_{2}$ = 17, with a percentage of 35%.

It can be seen that in some cases, analytical parameters including profile deviations and pitch deviations can be extracted from the double-flank measurements, thus providing a basis for the analysis and evaluation of process errors in gear manufacturing.

**Figure 6:**
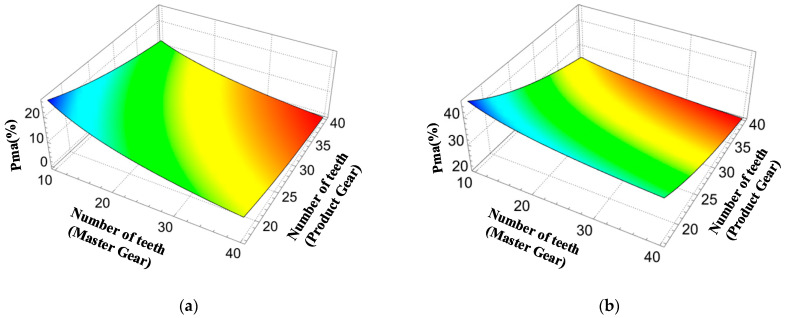
Relationship between the number of teeth and the proportion of measurable area for transverse pressure angles of 15° and 25°. (**a**) $\alpha_{0}=15{}^{\circ}$. (**b**) $\alpha_{0}=25{}^{\circ}$.

Further, in order to investigate the effect of pressure angle variation on the size of the measurable area, analyses were conducted at pressure angles of 15° and 25°, and the results are shown in Figure 6. It can be seen that the larger the transverse pressure angle, the greater the percentage of the measurable area in the profile.

**Figure 7:**
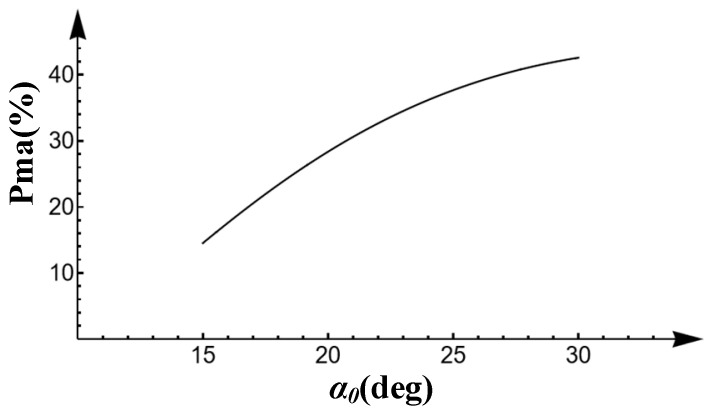
Proportion of measurable area for transverse pressure angle from 15° to 30° (*z*_1_ = 19, *z*_2_ = 20).

Under the condition of keeping the number of teeth of the product gear and the master gear at 19 and 20 (the number of teeth in Table 1), respectively, changing the transverse pressure angle of the two gears from 15° to 30° results in the change curve of $P_{\mathrm{ma}}$ as shown in Figure 7. As the transverse pressure angle becomes larger, the proportion of the measurable area to the whole profile area also becomes larger, which is a nonlinear relationship.

## 3. Experiments and Analysis

In order to verify the measurability of the analytical parameters in the measurable area in the double-flank measurement, various typical profile deviations were used as the profile deviations for the *i* − 1th, *i*th, and *i* + 1th tooth. Based on the gear parameters in Table 1 and the principle of the double-flank rolling tester in Figure 1, the simulation of the double-flank measurement results was conducted. Furthermore, the information on the center distance variation and the two angles from the double-flank measurement results was utilized to solve the analytical parameters [21].

### 3.1. Typical Profile Deviations

**Figure 8:**
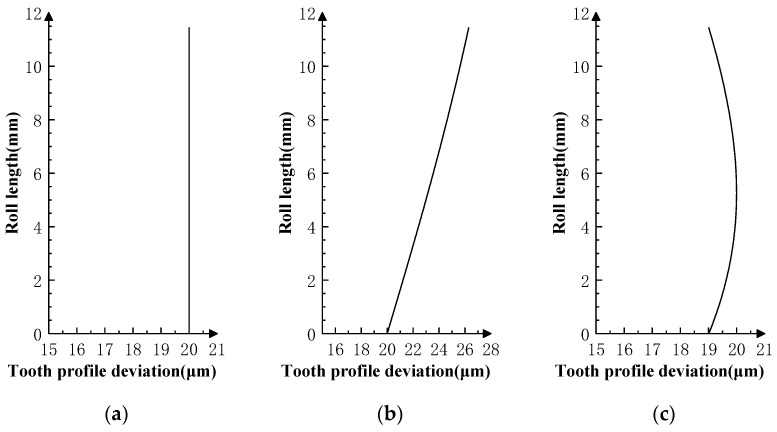

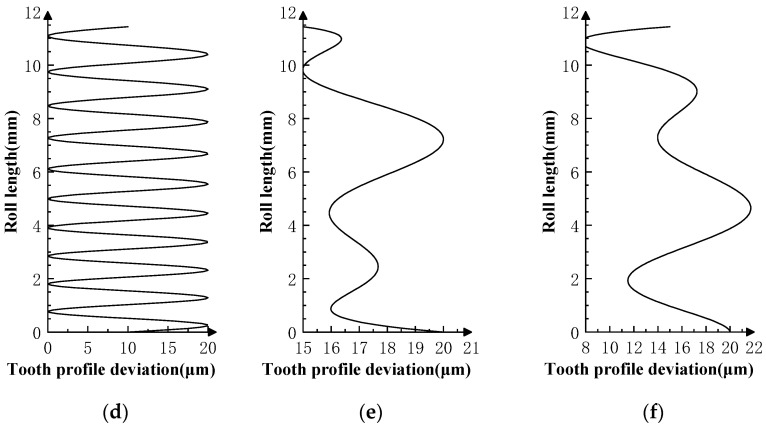
Type of profile deviations for simulations. (**a**) Zero-order error curve. (**b**) First-order error curve. (**c**) Second-order parabolic error curve. (**d**) Higher-order sinusoidal error curve. (**e**) Spline-type error curve Ⅰ. (**f**) Spline-type error curve Ⅱ.

Typical profile deviations can be categorized into zero-order translation error (Figure 8a), first-order inclination error (Figure 8b), second-order parabolic error (Figure 8c), and higher-order sinusoidal error (Figure 8d). Superposition of the profile deviations of each order can produce a spline-type error curve close to the actual gear profile deviations in Figure 8e,f.

### 3.2. Results and Analysis

**Figure 9:**
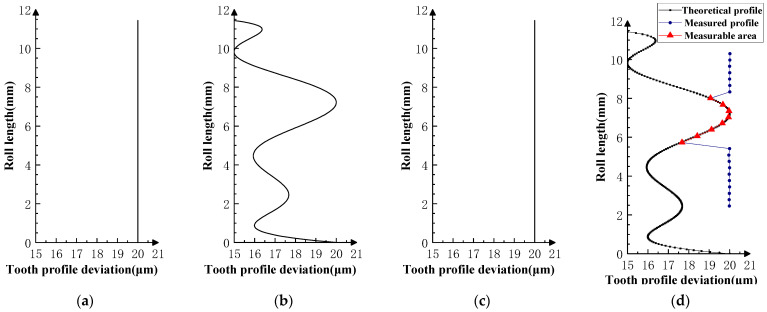
Experiment Ⅰ (zero-order error for front and rear teeth). (**a**) Theoretical profile error of *i* − 1th teeth. (**b**) Theoretical profile error of *i*th teeth. (**c**) Theoretical profile error of *i* + 1th teeth. (**d**) Profile measurement results and measurable area.

**Figure 10:**
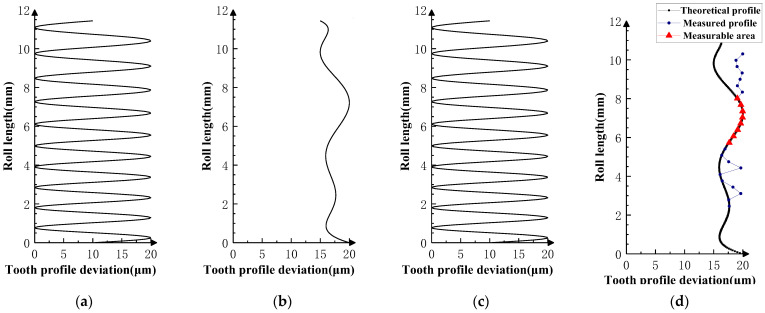
Experiment Ⅱ (higher-order error for front and rear teeth). (**a**) Theoretical profile error of *i* − 1th teeth. (**b**) Theoretical profile error of *i*th teeth. (**c**) Theoretical profile error of *i* + 1th teeth. (**d**) Profile measurement results and measurable area.

**Figure 11:**
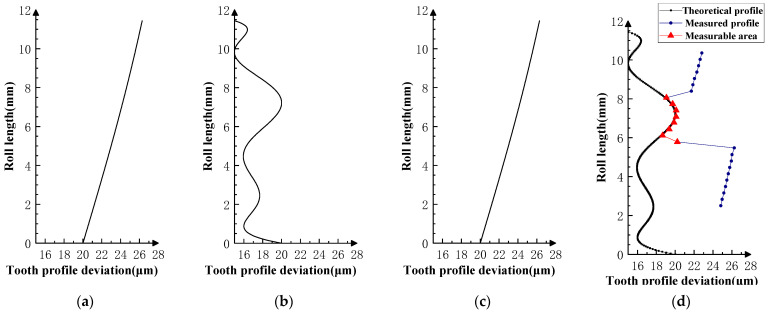
Experiment Ⅲ-1 (the *i* − 1st tooth is a first-order increasing error; the *i* + 1st tooth is a first-order increasing error). (**a**) Theoretical profile error of *i* − 1th teeth. (**b**) Theoretical profile error of *i*th teeth. (**c**) Theoretical profile error of *i* + 1th teeth. (**d**) Profile measurement results and measurable area.

**Figure 12:**
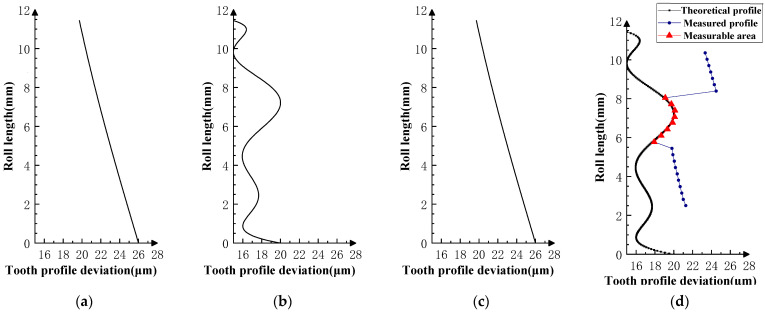
Experiment Ⅲ-2 (the *i* − 1st tooth is a first-order decreasing error, and the *i* + 1st tooth is a first-order decreasing error). (**a**) Theoretical profile error of *i* − 1th teeth. (**b**) Theoretical profile error of *i*th teeth. (**c**) Theoretical profile error of *i* + 1th teeth. (**d**) Profile measurement results and measurable area.

**Figure 13:**
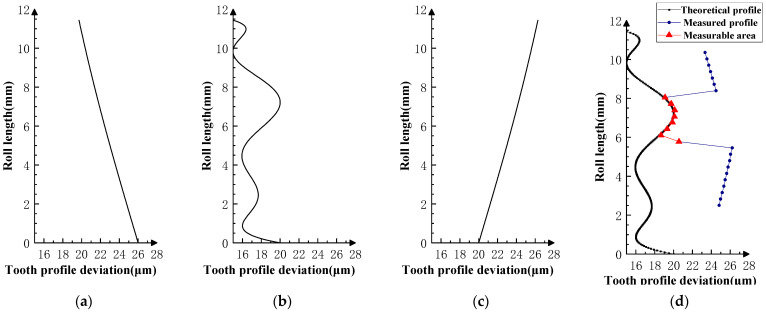
Experiment Ⅲ-3 (the *i* − 1st tooth is a first-order increasing error, and the *i* + 1st tooth is a first-order decreasing error). (**a**) Theoretical profile error of *i* − 1th teeth. (**b**) Theoretical profile error of *i*th teeth. (**c**) Theoretical profile error of *i* + 1th teeth. (**d**) Profile measurement results and measurable area.

**Figure 14:**
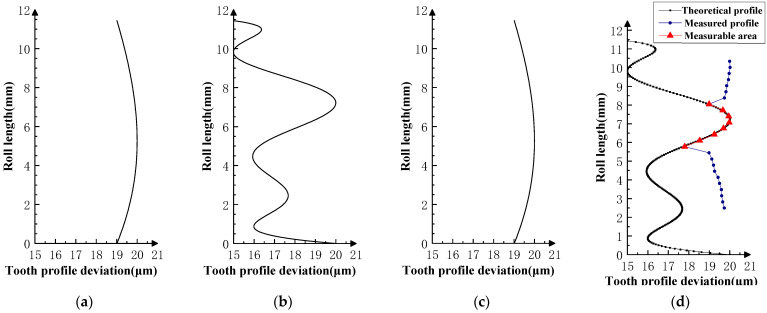
Experiment Ⅳ-1 (the *i* − 1st and *i* + 1st teeth are second-order errors, and the *i*-th tooth is the mid-convex spline curve error). (**a**) Theoretical profile error of *i* − 1th teeth. (**b**) Theoretical profile error of *i*th teeth. (**c**) Theoretical profile error of *i* + 1th teeth. (**d**) Profile measurement results and measurable area.

**Figure 15:**
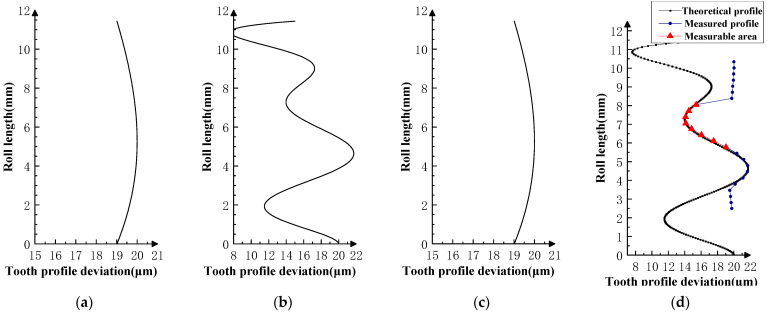
Experiment Ⅳ-2 (the *i* − 1st and *i* + 1st teeth are second-order errors, and the *i*-th tooth is the mid-concave spline curve error). (**a**) Theoretical profile error of *i* − 1th teeth. (**b**) Theoretical profile error of *i*th teeth. (**c**) Theoretical profile error of *i* + 1th teeth. (**d**) Profile measurement results and measurable area.

**Figure 16:**
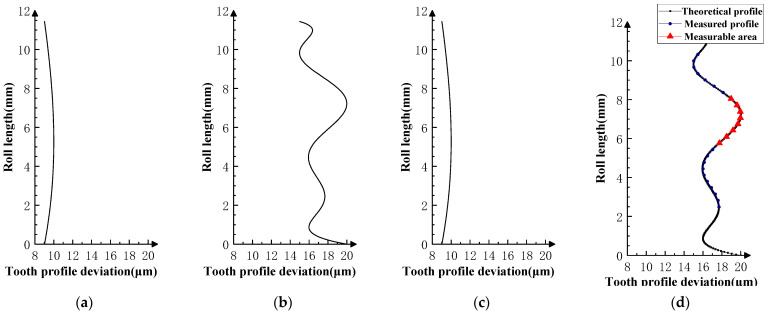
Experiment Ⅴ (the *i* − 1st and *i* + 1st teeth are small magnitude second-order errors, and the *i*-th tooth is the mid-convex spline curve error). (**a**) Theoretical profile error of *i* − 1th teeth. (**b**) Theoretical profile error of *i*th teeth. (**c**) Theoretical profile error of *i* + 1th teeth. (**d**) Profile measurement results and measurable area.

**Figure 17:**
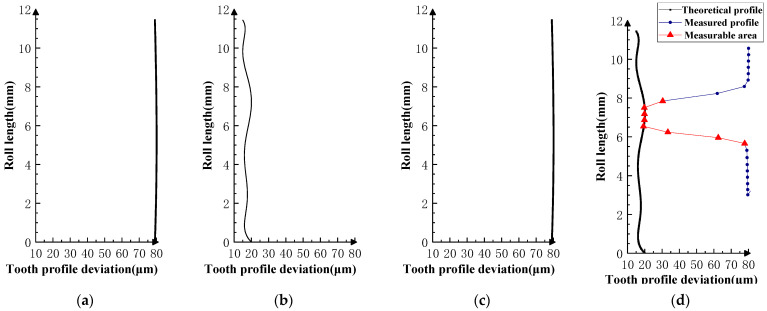
Experiment Ⅵ-1 (the *i* − 1st and *i* + 1st teeth are large-value second-order errors, and the *i*-th tooth is the mid-convex spline curve error). (**a**) Theoretical profile error of *i* − 1th teeth. (**b**) Theoretical profile error of *i*th teeth. (**c**) Theoretical profile error of *i* + 1th teeth. (**d**) Profile measurement results and measurable area.

**Figure 18:**
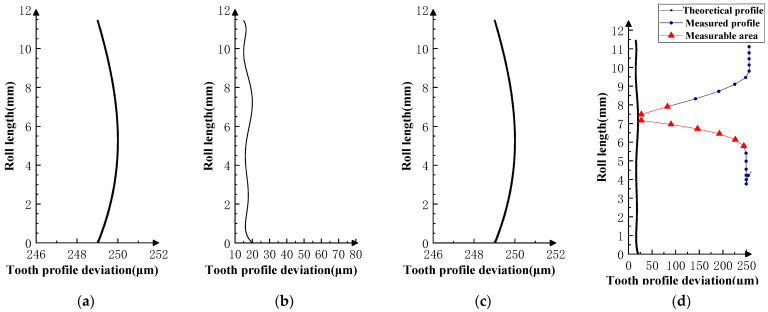
Experiment Ⅵ-2 (the *i* − 1st and *i* + 1st teeth are larger-value second-order errors, and the *i*-th tooth is the mid-convex spline curve error). (**a**) Theoretical profile error of *i* − 1th teeth. (**b**) Theoretical profile error of *i*th teeth. (**c**) Theoretical profile error of *i* + 1th teeth. (**d**) Profile measurement results and measurable area.

Various typical profile deviations were superimposed on three successive corresponding flanks of the product gear, and several sets of tests were carried out. The theoretical profile deviations for the three teeth of *i* − 1th, *i*th, and *i* + 1th, together with the measurability test results, are shown in Figure 9, Figure 10, Figure 11, Figure 12, Figure 13, Figure 14, Figure 15, Figure 16, Figure 17 and Figure 18. With the exception of Experiments V and VI, the magnitude of the profile deviations corresponds to the tolerance class 9 defined in ISO 1328-1 [22].

In Experiment I, the front and rear teeth are with zero-order errors, while the *i*th tooth is with a spline error. In Experiment II, the front and rear teeth are with higher-order sinusoidal errors. In Experiment III, the front and rear teeth are with first-order errors. Experiment III was divided into three situations: the trend of front and rear tooth error increasing simultaneously, decreasing simultaneously, and having different trends. In Experiment IV, the front and rear teeth are with parabolic errors, and the *i*th tooth is with two types of spline errors, convex and concave.

The profile deviations of tooth *i* − 1th and tooth *i* + 1th in Experiment Ⅰ, Ⅱ, Ⅲ, and Ⅳ are greater than or equal to that of tooth *i*th, but the magnitudes of the errors are basically equivalent, as shown in Figure 9, Figure 10, Figure 11, Figure 12 and Figure 13. The measured profile deviations of the product gear are basically equal to the theoretical profile deviations of the tooth *i*th only in the measurable area, which verifies the measurability of the analytical parameters in the measurable area.

As shown in Figure 11, Figure 12 and Figure 13, three sets of first-order error tests in Experiment III were conducted to investigate the effect of error on the measurement results. Among them, the measured profile deviations of the addendum and dedendum meshing zones of the *i*th tooth in the measurement results of Experiment Ⅲ-2 have different inclination directions, indicating that the test results conform to a hidden rule that the theoretical profile deviations of the *i* + 1th tooth mainly affect the dedendum meshing zone of the *i*th tooth, and the theoretical profile deviations of the *i* + 1th tooth mainly affect the addendum meshing zone of the *i*th tooth.

As shown in Figure 16, the profile deviations of the *i* − 1th and *i* + 1th teeth in Experiment V are smaller than that of the *i*th teeth, and the actual measurable area of the profile deviations is much larger than the theoretical measurable area, indicating that the measurable area can be enlarged by thinning the tooth flanks of the front and rear neighboring teeth of the master gear. This is similar to the role of the teeth-skipped gear (thinning gear) in the gear integrated error measurement [23,24,25,26].

As shown in Figure 17 and Figure 18, the profile deviations of the *i* − 1th and *i* + 1th teeth in Experiment VI are much larger than that of the *i*th tooth; as the profile deviations of the *i* − 1th and *i* + 1th teeth gradually increase, the overlap area between the measured profile deviations and the theoretical profile deviations of the ith tooth gradually decreases. This shows that if the profile deviations of the neighboring tooth of the measured tooth are too large (the amplitude of the error in VI-2 is as high as 250 µm), the profile deviations of the measured tooth will be impossible to measure in the double-flank measurement.

Through analysis of the experimental results, the size of the measurable area on the flank is related to the actual gear error. The type, magnitude, and shape of the error are factors that affect the size of the measurable area. Furthermore, some valuable assumptions can be made:

1. The size of the measurable area is mainly affected by the amplitude of the error (related to the accuracy grade of the gear); the larger the error and the lower the accuracy grade, the smaller the measurable area will be. However, when the gear accuracy is equal to or better than the medium accuracy level (grade 5–7 in ISO 1328-1), the size of the measurable area is basically determined by geometric factors.
2. When the profile deviations of the front and rear teeth are much larger than that of the measured flank, the measurable area will be smaller, as shown in Experiment Ⅵ (Figure 17 and Figure 18).
3. When the profile deviations of the front and rear are smaller than that of the measured flank, the measurable area becomes larger. This is shown in Experiment V (Figure 16).
4. The size of the measurable area is basically independent of the type of profile deviations. Under the same amplitude conditions, different profile deviation types have no significant effect on the size of the measurable area.
5. The shape of the error of the same accuracy grade has no significant effect on the size of the measurable area. As in Experiment Ⅳ (Figure 14 and Figure 15), the profile deviations of the measured tooth have two types of shapes in the measurable area, convex and concave, and there is no obvious change in the size of the measurable area under the two shapes.

## 4. Conclusions

This paper analyzes the measurability of analytical parameters in gear double-flank measurement. The definition of the measurable area of the analytical parameters in the double-flank measurement is presented, the calculation method of the size and position of the measurable area is proposed, the rule of the change in the size of the measurable area with the number of teeth and the pressure angle of the meshing gears is calculated, and the effect of the gear error on the measurable area is analyzed.

The experimental data show that the measurable area exists when the accuracy of product gear is class 9 or higher and when there are zero-order error, first-order error, second-order error, higher-order error, and spline error on the profile of the product gear.

The higher the accuracy of the product gear, the more stable the measurable area of the analytical parameters will be. When the product gear is of medium or higher accuracy (e.g., automotive gear), it is possible to accurately isolate the profile deviations of the measurable area based on the results of the double-flank measurement and then obtain the pitch deviations, radial runout, and so on.

This study of the measurability of the analytical parameters in double-flank measurement is based on a cylindrical spur gear pair. Similar studies can be carried out in the future on other types of gears, such as helical gears. In addition, the size of the measurable area is affected by the actual gear accuracy. The influence rules of gear deviations, such as pitch and helix deviations, are complicated and not discussed in this paper for the time being. The influence rules require further research in the future.

## Figures and Tables

**Table 1:** Gear parameters in the experiments.

Parameter	Modulus (mm)	Number of Teeth	Transverse Pressure Angle (eg)	Profile Shift Coefficient
Product gear	2	20	20	0
Master gear	2	19	20	0

## Data Availability

The data that support the findings of this study are available from the corresponding author upon reasonable request.
